# Early Differential Diagnosis of Epstein–Barr Virus-Associated Hemophagocytic Lymphohistiocytosis and Macrophage Activation Syndrome in Children: A Clinical Prediction Model Based on 106 Patients

**DOI:** 10.3390/pathogens15080828

**Published:** 2026-08-06

**Authors:** Mengjia Pei, Yuewen Su, Jiaying Ding, Weifang Zhou, Chu Chu

**Affiliations:** Department of Infectious Diseases, Children’s Hospital of Soochow University, Suzhou 215006, China; pmj2026@163.com (M.P.); suyuewen0314@163.com (Y.S.); djy18761950382@163.com (J.D.)

**Keywords:** Epstein–Barr virus, rheumatology, hemophagocytic lymphohistiocytosis, early diagnosis, model

## Abstract

The study screened clinical and laboratory indicators accessible within 48 h of admission for children with Epstein–Barr virus-associated hemophagocytic lymphohistiocytosis (EBV-HLH) and macrophage activation syndrome (MAS) to establish an efficient and convenient differential diagnosis model. The study retrospectively analyzed the clinical data and laboratory results obtained within 48 h of admission from 106 pediatric patients initially hospitalized and diagnosed with EBV-HLH or MAS at the Children’s Hospital of Soochow University from January 2019 to November 2024. Univariate logistic regression and LASSO regression filtered the variables alongside cross-validation and dimensionality reduction. The final regression model constructed a predictive nomogram, calibration curve, ROC curve, and DCA curve to evaluate the discrimination capacity and net clinical benefit of the model. Univariate logistic regression, LASSO regression with five-fold cross-validation, and stepwise multivariate logistic regression identified skin rash, bone marrow hemophagocytosis, fibrinogen, neutrophil percentage, and hepatosplenomegaly as the core predictors for differentiating EBV-HLH from MAS. The constructed nomogram and diagnostic calculator demonstrated favorable discriminative ability, with an area under the receiver operating characteristic curve of 0.938 (95% CI: 0.894–0.974). The calibration curve showed good agreement between predicted probabilities and actual observed outcomes, yielding a Brier score of 0.099. Decision curve analysis indicated that the model provided a significant positive net clinical benefit across a risk threshold range of 5% to 95%. A simple scoring system based on common clinical indicators effectively distinguished EBV-HLH from immune-related HLH in the early stages and guided initial treatment decisions.

## 1. Introduction

Pediatric hemophagocytic lymphohistiocytosis (HLH) is an acute and severe clinical condition with a high mortality rate [1]. Among its various etiologies, EBV-associated HLH (EBV-HLH) and macrophage activation syndrome (MAS) represent the two most common types in clinical practice [2]. In the early stages of onset, these two conditions often present with highly overlapping systemic inflammatory responses, including persistent high fever, hepatosplenomegaly, and cytopenia, making them highly confusing [3]. However, their underlying pathological mechanisms and preferred treatment plans differ significantly, as EBV-HLH management focuses on controlling the primary infection alongside chemotherapy [4], whereas MAS requires immunosuppressive therapy targeting the underlying rheumatic disease [5]. Therefore, the accurate and early differentiation of these two etiologies directly relates to the selection of treatment strategies and the survival prognosis of pediatric patients. Although recent studies attempt to develop prediction tools for severe illness or mortality risk using logistic regression or machine learning algorithms [6,7], most of these models focus on determining patient survival prognosis or treatment response. There remains a lack of comprehensive prediction models specifically designed to differentiate between EBV and rheumatic etiologies based on routine laboratory indicators. Furthermore, the few existing clinical models often rely on complex mathematical formulas and fail to translate into bedside tools for direct use by physicians, which limits their application in actual clinical practice. To address the limitations of current research, this study constructed a routine indicator prediction model specifically designed for the early differentiation of EBV and rheumatic etiologies following a pediatric HLH diagnosis. Based on real clinical data, we identified the core diagnostic indicators and developed a simple online diagnostic prediction calculator. This tool converts complex prediction models into intuitive probability values, which helps physicians quickly assess the etiology within 48 h of admission and seize the window for early treatment.

## 2. Materials and Methods

### 2.1. Study Population

The study collected clinical data from pediatric patients initially hospitalized and definitively diagnosed with EBV-HLH or MAS at the Children’s Hospital of Soochow University between January 2019 and November 2024. The patients were divided into an EBV-HLH group (*n* = 49) and an MAS group (*n* = 57). The EBV-HLH group comprised 22 males and 27 females, whereas the MAS group consisted of 29 males and 28 females, indicating no significant gender difference between the two groups (*p* > 0.05). The median age of children in the EBV-HLH group was 5.17 (2.92–7.33) years, the mean age was 5.51 years, and the median age in the MAS group was 7.50 (3.75–12.92) years and the mean age was 8.03 years, which presented a statistically significant difference between the two groups (*p* < 0.05).

### 2.2. Diagnostic Criteria

The diagnostic criteria for EBV-HLH and MAS followed the HLH-2004 guidelines developed by the Histiocyte Society [8] and the 2016 macrophage activation syndrome (MAS) classification criteria jointly issued by EULAR, ACR, and PRINTO [9]. Evidence of EBV infection aligned with the expert consensus on the diagnosis and treatment principles of EBV infection-related diseases in children [10]. All patients represented initial diagnostic admissions at this hospital. The study excluded cases with extensive missing clinical data, HLH of unknown etiology, and primary HLH.

### 2.3. Study Methods

The research collected clinical symptoms, signs, and laboratory test results of the study subjects. These laboratory results encompassed routine blood tests, coagulation profiles, ferritin levels, comprehensive metabolic panels, humoral immunity, and lymphocyte subsets. The children were classified into the EBV-HLH group and the MAS group according to their admission diagnoses.

### 2.4. Statistical Analysis

Statistical analyses were conducted using Python (3.12) and R software (4.5.2), and the manuscript was polished using Gemini (3.1 pro). Multiple imputation handled missing data, and continuous variables initially underwent the Shapiro–Wilk normality test. Continuous variables were evaluated using the *t*-test (if normally distributed) or the Mann–Whitney U test (if non-normally distributed). Normally distributed data were expressed as mean ± standard deviation, whereas non-normally distributed data were presented as median (P25–P75). Categorical variables were assessed primarily through the chi-square test based on expected frequencies, or Fisher’s exact test when data distributions were sparse. Because age exhibited a statistically significant difference between groups, skewed continuous variables underwent logarithmic transformation prior to traditional analysis of covariance (ANCOVA) with age as a covariate, whereas non-normally distributed variables unsuitable for log transformation underwent rank-based ANCOVA for age adjustment [11]. Univariate logistic regression initially screened all variables. Variables showing *p* < 0.15 in the preliminary screening entered least absolute shrinkage and selection operator (LASSO) regression, which applied 5-fold cross-validation to determine the optimal penalty coefficient and achieved high-dimensional variable reduction. Subsequently, multivariable logistic regression incorporated the LASSO-selected variables to identify independent predictors for patients with EBV-HLH. Based on the final regression model, the study constructed a predictive nomogram and developed a web-based dynamic interactive diagnostic prediction calculator using HTML, CSS, and JavaScript. Receiver operating characteristic (ROC) curves evaluated the discrimination capability of the model, while calibration curves combined with Brier scores assessed probability calibration. Internal validation employed bootstrapping with 1000 resamples to generate calibration curves for evaluating model consistency, and the Hosmer–Lemeshow goodness-of-fit test quantified model calibration, where *p* > 0.05 indicated a satisfactory fit without significant bias between predicted and actual incidence rates. Decision curve analysis (DCA) evaluated the net clinical benefit of the model.

## 3. Results

### 3.1. Clinical Manifestations and Signs in the EBV-HLH and MAS Groups

Fever served as a common manifestation among the 106 pediatric patients. Children in the EBV-HLH group exhibited hepatosplenomegaly and bleeding more frequently than those in the MAS group. Conversely, children in the MAS group presented with rash more often than those in the EBV-HLH group. These differences were statistically significant (*p* < 0.05), as detailed in Table 1.

### 3.2. Comparison of Laboratory Test Results Between the EBV-HLH and MAS Groups

#### 3.2.1. Comparison of Routine Blood, Biochemical, and Other Indicators Between the EBV-HLH and MAS Groups

Pediatric patients in the EBV-HLH group presented with lower levels of WBC, NEU, HB, PLT, FIB, and TC than those in the MAS group. Conversely, levels of LYM, D-dimer, AST, GGT, and LDH were higher in the EBV-HLH group than in the MAS group. These differences were statistically significant (*p* < 0.05). After adjusting for age, both FER and CRP reached statistical significance, whereas HB became statistically insignificant, as shown in Table 2.

#### 3.2.2. Comparison of Immune Indicators Between the EBV-HLH and MAS Groups

Pediatric patients in the EBV-HLH group demonstrated higher levels of C4, CD3+%, CD3+CD8+%, and LDH than those in the MAS group. Conversely, the CD4+/CD8+ ratio, CD3-CD19+%, CD19+CD23+%, and CD3CD19+ cell counts were lower in the EBV-HLH group. These differences were statistically significant (*p* < 0.05). After adjusting for age, CD3+CD4+%, CD3+CD4+, and IgA achieved statistical significance, whereas CD4+/CD8+ became statistically insignificant, as presented in Table 3.

### 3.3. Univariate Logistic Regression and LASSO Regression

**Figure 1:**
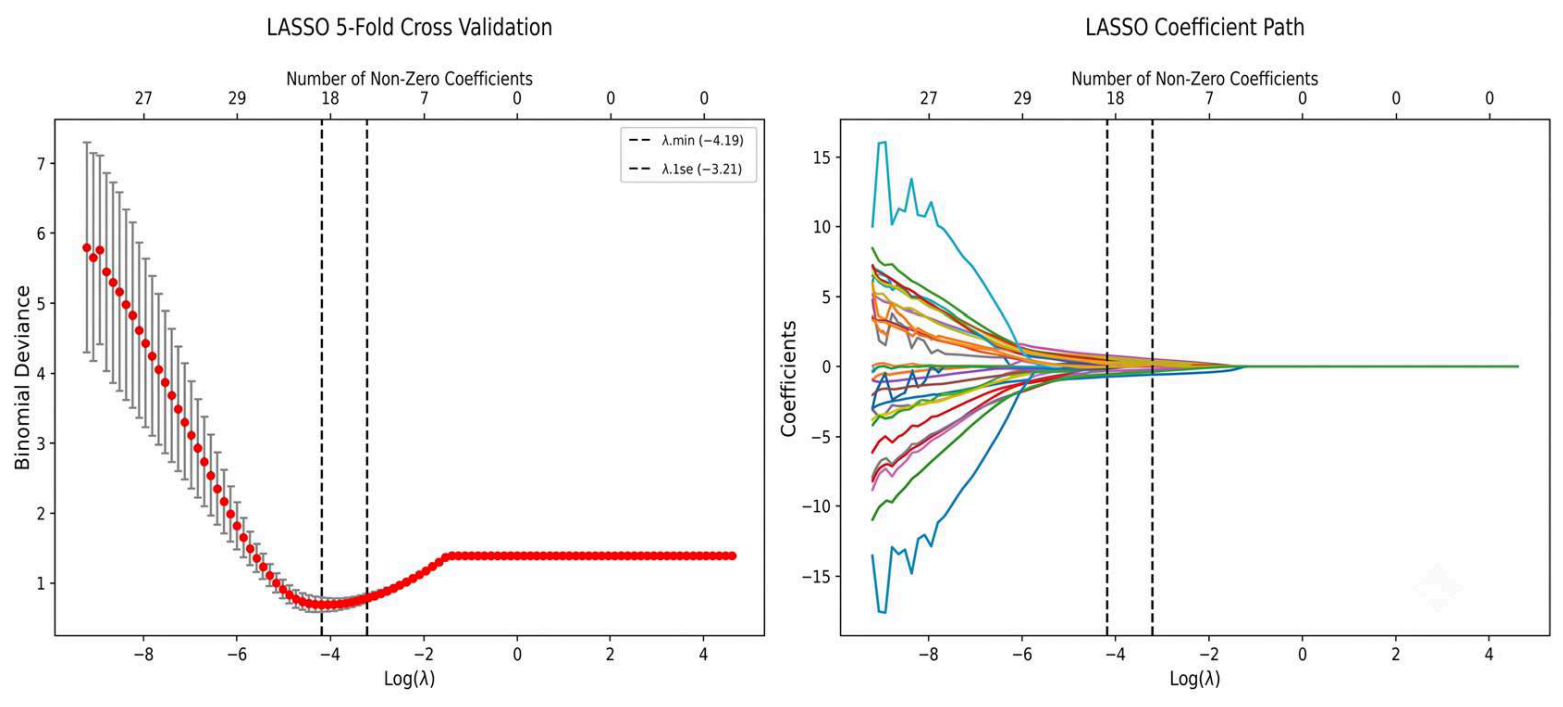
LASSO regression coefficient profiles and cross-validation plot.

Univariate logistic regression initially screened the aforementioned variables and identified 33 indicators with a *p* value < 0.15, as presented in Table 4. These variables subsequently entered the LASSO regression utilizing 5-fold cross-validation to generate the LASSO regression coefficient profile and cross-validation plot, as illustrated in Figure 1. The selection of log(λ) = −3.21 as the cutoff criterion yielded 15 feature variables excluding age. Collinearity diagnostics using the variance inflation factor for these 15 variables revealed that all variance inflation factor values were below 2.5, indicating an absence of significant multicollinearity. Considering the time-sensitivity of clinical decision-making in pediatric critical care and the principle of model simplicity, a pediatric multidisciplinary team conducted a cross-review. While ensuring comprehensive coverage of underlying pathological mechanisms including the coagulation system, hematopoietic system, immune inflammation, and histopathology, the study further distilled these variables into five core indicators. These core indicators comprised hepatosplenomegaly as a highly specific feature of EBV-HLH, rash as a highly specific feature of MAS, bone marrow hemophagocytosis representing the histopathological gold standard, fibrinogen indicating excessive coagulation and inflammatory consumption, and neutrophil percentage representing the hematopoietic system and routine blood parameters.

The cross-validation plot demonstrates the optimization of λ through 5-fold cross-validation. The lower x-axis represents log(λ), and lower y-axis values indicate an optimal model goodness-of-fit. The upper x-axis denotes the number of predictor variables retained in the model at various regularization strengths. Two vertical dashed lines mark statistically significant critical λ values. The optimal λ located one standard error from the minimum deviance, known as lambda.1se, is preferred and indicated by the right dashed line.

The LASSO regression coefficient profile illustrates the dynamic variation of variable coefficients. The y-axis represents the estimated coefficient values, the lower x-axis utilizes a log(λ) scale, and the upper x-axis corresponds to the number of non-zero coefficients within the model. As the value of log(λ) increases, the regression coefficients of all variables exhibit a gradual shrinkage trend before eventually stabilizing.

### 3.4. Multivariable Logistic Regression

To construct the final diagnostic model, the study incorporated five core clinical indicators, namely rash, hepatosplenomegaly, FIB, NEU, and bone marrow hemophagocytosis. Additionally, because age represented a potential confounding factor with statistical significance in univariate analysis, it entered the final multivariable logistic regression model. The model yielded an EPV = 8.17. Although this value fell slightly below the traditionally recommended threshold of 10, the overfitting risk of the multivariable model remained within an acceptable range, and the model exhibited strong mathematical stability. This stability is supported by the prior application of LASSO regression for strict variable penalty and dimensionality reduction, as well as the fact that all included variables exhibited a VIF < 1.5. See Table 5.

### 3.5. ROC Curves, DCA Curves, Calibration Curves, Nomogram, and Diagnostic Prediction Calculator

**Figure 2:**
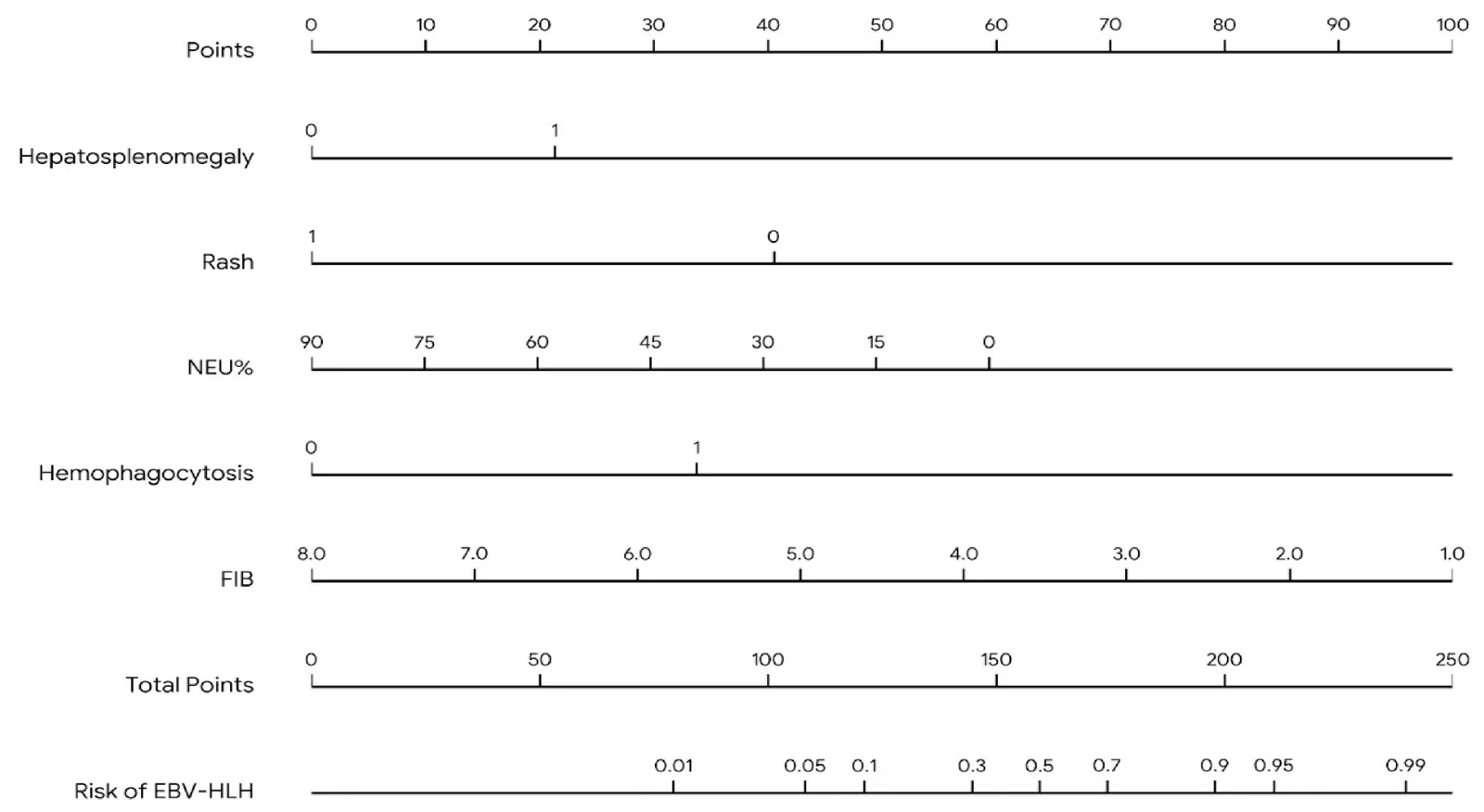
Nomogram for EBV-HLH prediction.

**Figure 3:**
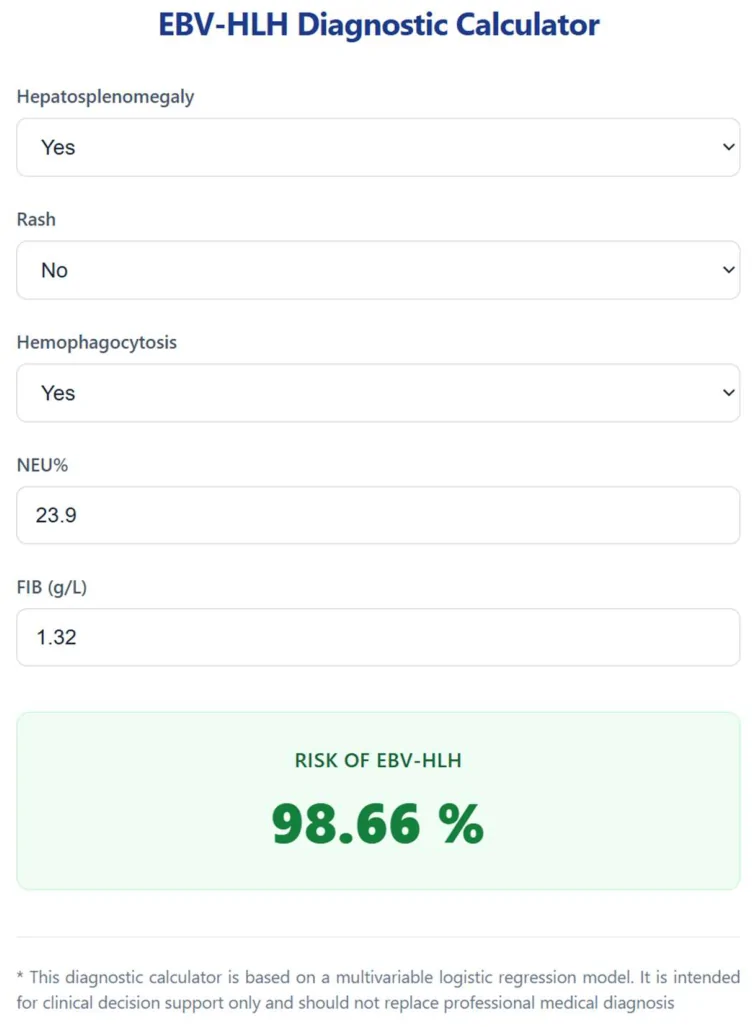
Example of the diagnostic prediction calculator using data from one pediatric patient. * This diagnostic calculator is based on a multivariable logistic regression model. It is intended for clinical decision support only and should not replace professional medical diagnosis.

**Figure 4:**
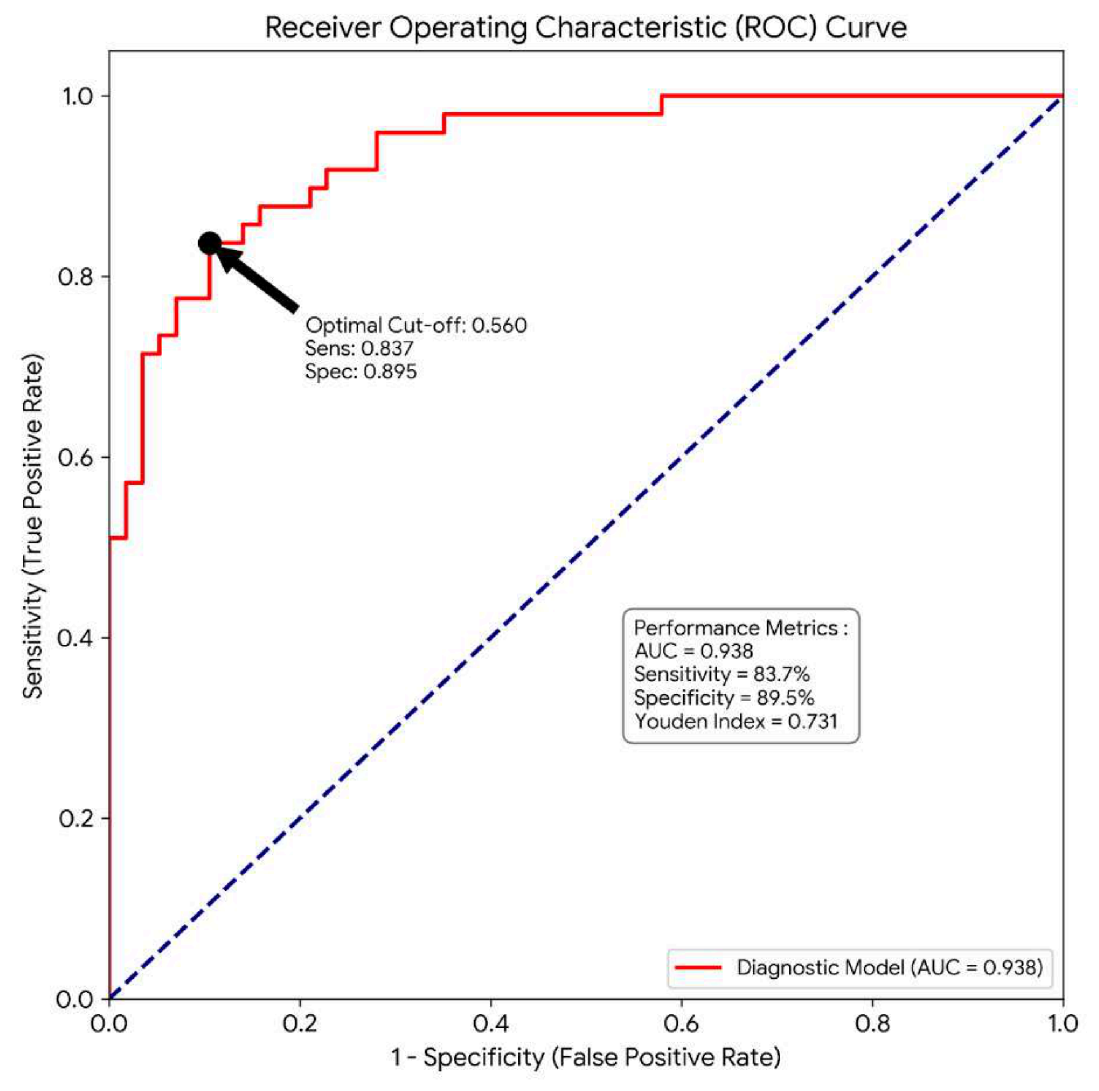
ROC curve.

**Figure 5:**
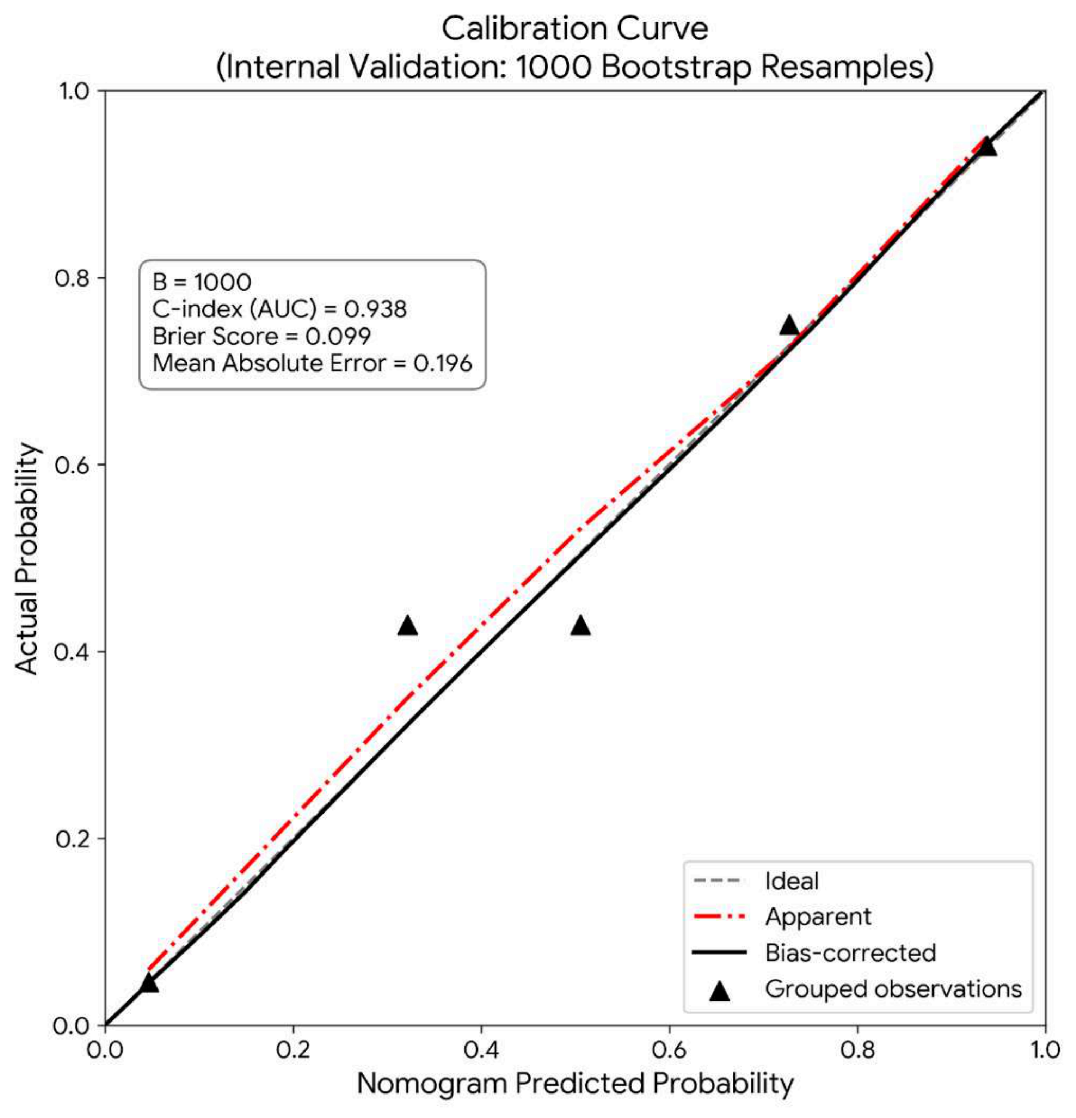
Calibration curve.

**Figure 6:**
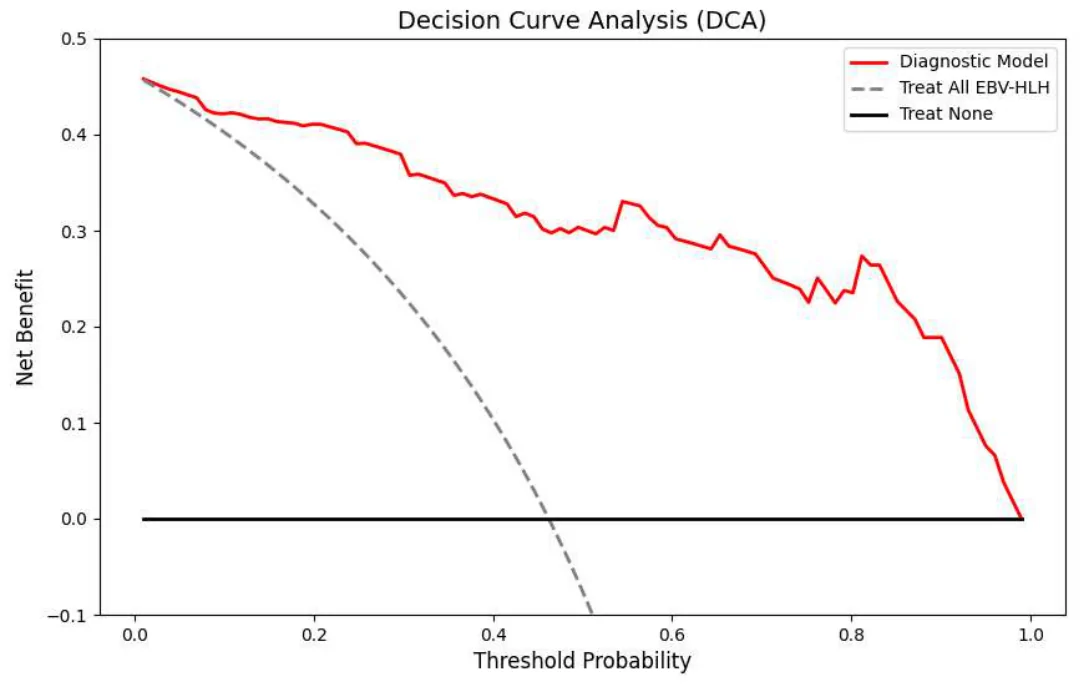
DCA curve.

Although hepatosplenomegaly did not reach statistical significance due to sample size limitations, its OR reached 3.440, and as a core clinical sign of EBV-HLH, it was crucial for improving the clinical interpretability of the model; therefore, it was retained. Conversely, age itself had no statistically significant independent predictive effect in differentiating EBV-HLH, so it was excluded from the model. To construct a more streamlined early diagnostic tool that is more conducive to practical pediatric clinical operations, this study refitted a five-variable multivariable regression model after excluding age. Based on this simplified model, a visual diagnostic nomogram (see Figure 2) and a diagnostic prediction calculator (see Figure 3) were constructed to quantify the probability of an individual patient being diagnosed with EBV-HLH. Receiver operating characteristic (ROC) curve analysis (see Figure 4 and Table 6) showed that the model AUC value was 0.938, which indicates that the model possesses excellent predictive ability. The calibration curve (see Figure 5) demonstrated that the nomogram model did not exhibit a severe overfitting tendency, and its predicted disease probability showed extremely high consistency with the actual occurrence. Decision curve analysis (see Figure 6) further confirmed the clinical utility of the model. Furthermore, to evaluate the clinical utility of the model, we calculated the predictive values based on the cohort prevalence (46.2%). The model demonstrated a Positive Predictive Value (PPV) of 87.2% and a Negative Predictive Value (NPV) of 86.4%, indicating a high probability of correct classification for both positive and negative predictions.

In this nomogram, a specific value for each predictive indicator corresponds to a score on the top Points axis. Summing the individual scores of all indicators for a patient yields the Total Points. Projecting this total score downward reveals the predicted probability of the patient developing EBV-HLH.

To facilitate rapid bedside application of this model by pediatric clinicians, the study converts the weights of the five aforementioned core predictors into an online calculator available at https://adorable-griffin-811904.netlify.app/ (accessed on 4 August 2026). Clinicians simply input the clinical and laboratory indicators obtained within 48 h of admission via a mobile device or computer, and the system automatically generates the predicted probability of the patient having EBV-HLH. This process simplifies the manual calculation steps required by traditional nomograms, as illustrated in Figure 3.

The results indicate that the combined prediction model comprising five core variables demonstrates strong discriminatory capacity. The area under the curve (AUC) of the final model reaches 0.938 (95% CI: 0.893 to 0.973), significantly outperforming any single predictive indicator, where individual AUC values range from 0.718 to 0.788. Based on the Youden index of 0.731, the optimal probability cutoff value for predicting EBV-HLH is 0.560. At this threshold, the model yields a sensitivity of 83.7% and a specificity of 89.5%.

Internal validation employs 1000 bootstrap resamples. Following optimism correction, the final average Brier score stabilizes at 0.099, indicating minimal prediction error. The calibration curve demonstrates that the bias-corrected predicted probability curve aligns closely with the ideal diagonal line representing perfect prediction, resulting in a mean absolute error of only 0.196. Furthermore, the Hosmer–Lemeshow goodness-of-fit test confirms no statistically significant difference between the predicted and actual observed values (X^2^ = 4.568, *p* = 0.802). This result quantitatively shows that the diagnostic model exhibits no significant overestimation or underestimation and possesses excellent calibration capability and goodness of fit.

The results demonstrate that the net clinical benefit of applying this nomogram model to support diagnostic decisions consistently and significantly outperforms the extreme strategies of treating all patients for EBV-HLH or treating no patients, where treating no patients equates to assuming all cases are MAS.

## 4. Discussion

The differences in clinical signs between EBV-HLH and MAS reflect their distinct pathophysiological processes. This study found that the incidence of hepatosplenomegaly in the EBV-HLH group was significantly higher than that in the MAS group, whereas rash is more common among pediatric patients in the MAS group. This likely occurs because EBV directly infects T cells, NK cells, or B cells, which leads to the excessive proliferation and infiltration of abnormally activated lymphocytes and macrophages within the reticuloendothelial system, including the liver and spleen, thereby causing significant organ enlargement [12]. Simultaneously, the infiltrating cells cause hepatocyte damage, which aligns with the higher AST, GGT, and LDH levels observed in the EBV-HLH group in this study [13]. Conversely, MAS typically occurs secondary to systemic autoimmune diseases such as systemic juvenile idiopathic arthritis (sJIA), and the intense systemic autoinflammatory response frequently accompanies the characteristic rash of the primary disease [14].

In terms of hematopoietic system impairment, bone marrow hemophagocytosis and the neutrophil percentage (NEU%) exhibit significant differences between the two groups. Overactivated macrophages engulf various formed elements in the blood to cause hemophagocytosis, which accelerates blood cell destruction [15]. In this study, the EBV-HLH group showed a significantly higher proportion of early bone marrow hemophagocytosis than the MAS group, and it demonstrated more pronounced decreases in WBC, NEU%, and PLT. The cytokine storm triggered by EBV infection is likely more direct and intense, exerting a stronger inhibitory effect on the proliferation and differentiation of bone marrow hematopoietic stem cells, which leads to evident peripheral pancytopenia and bone marrow hemophagocytosis in the early stages [16]. In contrast, during the early stages of MAS, the primary systemic inflammatory response, such as sJIA, typically manifests as leukocytosis and an elevated neutrophil proportion, and progressive decreases in leukocytes and platelets only emerge gradually as the disease advances.

Coagulopathy represents a crucial pathological feature in pediatric patients with HLH. Fibrinogen (FIB) functions not only as a vital coagulation factor but also as an acute-phase reactant [17]. The results of this study indicated that pediatric patients in the EBV-HLH group exhibited significantly lower FIB levels than those in the MAS group. Two mechanisms primarily account for this finding. First, severe liver injury induced by EBV-HLH significantly impairs the hepatic synthesis of fibrinogen. Second, excessive inflammatory responses activate the coagulation and fibrinolytic systems, leading to massive fibrinogen consumption and elevated D-dimer levels, an observation consistent with the significantly higher D-dimer levels found in the EBV-HLH group in this study [18,19]. Conversely, during the early stages of MAS, acute inflammatory stimulation often causes FIB to either compensatorily increase or remain normal as an acute-phase protein, and a consumptive decline occurs only during the peak phase of the disease [20].

Notably, this study adequately accounts for the influence of age as a physiological confounder on laboratory indicators. For example, after age adjustment, the difference in hemoglobin (HB) levels between the two groups becomes non-significant, with the *p* value changing from 0.025 to 0.153. This result indicates that the early difference in anemia between the two groups actually reflects a physiological phenomenon resulting from differing baseline ages. In contrast, classic HLH diagnostic indicators such as ferritin (FER) and immune indicators like CD3+CD4+% successfully reach statistical significance after eliminating age interference, as demonstrated by the *p* value of FER dropping from 0.059 to 0.024. This finding suggests that the age factor initially obscured the true efficacy of these indicators, which reaffirms the important clinical value of ferritin in the early etiological differentiation of HLH [21,22].

The early and accurate differentiation between EBV-HLH and MAS holds important clinical value for optimizing treatment strategies and improving patient outcomes. Although clinical practice currently utilizes the HLH-2004 and 2016 MAS classification criteria, rapidly distinguishing the etiology during the very early stages of the disease remains difficult. This study first used univariate logistic regression to screen candidate variables with significant specificity. To overcome potential multicollinearity in high-dimensional data and achieve effective dimensionality reduction, the study further applied LASSO regression combined with 5-fold cross-validation before introducing multivariable logistic regression. While ensuring coverage of the underlying pathological mechanisms and excluding age as a natural confounder, the analysis ultimately extracted five core indicators including hepatosplenomegaly, rash, bone marrow hemophagocytosis, fibrinogen, and neutrophil percentage. Based on these indicators, the study constructed a visual early differential diagnostic nomogram model and a diagnostic prediction calculator. This model demonstrated excellent discriminatory capability, with the area under the receiver operating characteristic curve reaching 0.938 (95% CI: 0.893–0.973). Following internal validation with 1000 bootstrap resamples, the calibration curve showed that the predicted probabilities aligned closely with the actual observed probabilities, the corrected Brier score was only 0.099, and the Hosmer–Lemeshow goodness-of-fit test confirmed the absence of significant bias in the model. Decision curve analysis indicated that the model provided a significant positive net clinical benefit.

In summary, this study constructed a clinical prediction model focusing on the early etiological differentiation of pediatric HLH and developed an online calculation tool based on this framework. Physicians only need to input the routine clinical and laboratory indicators obtained within 48 h of admission to rapidly receive a probability reference for the etiological tendency. For pediatric patients highly predicted to have EBV-HLH, this tool can serve as an early warning indicator, prompting clinicians to prioritize critical confirmatory diagnostics (such as EBV DNA viral load and genetic testing) and prepare for potential targeted therapies. Conversely, for patients trending toward MAS, the model provides adjunctive reference data that may assist clinicians in evaluating the risk–benefit ratio of early high-dose glucocorticoid and immunosuppressant administration. This study has several limitations that must be acknowledged. Primarily, this research is a single-center retrospective analysis with a limited sample size. Consequently, the current diagnostic tool must be regarded merely as a preliminary predictive model pending external validation. Furthermore, another significant limitation is that this study was conducted exclusively in China. Therefore, future prospective studies with large multicenter cohorts are strictly necessary to externally validate the model. Specifically, these data must be validated across different countries and diverse demographics to definitively confirm the broad clinical applicability and generalizability of the model.

## 5. Conclusions

A simple scoring system based on common clinical indicators effectively distinguishes EBV-HLH from immune-related HLH in the early stages and guides initial treatment decisions.

## Figures and Tables

**Table 1:** Comparison of clinical manifestations between the EBV-HLH and MAS groups [*n* (%) or median (P25–P75)].

Factor	EBV-HLH (*n* = 49)	MAS (*n* = 57)	X^2^/Z Value	*p* Value
Duration of fever	7.00 (7.00–7.00)	7.00 (4.00–7.00)	1.708	0.088
Hepatosplenomegaly	42 (85.7%)	17 (29.8%)	31.123	<0.001
Lymphadenopathy	39 (79.6%)	44 (77.2%)	0.004	0.950
Jaundice	6 (12.2%)	4 (7.0%)	—	0.508
Edema	21 (42.9%)	16 (28.1%)	1.927	0.165
Polyserositis	31 (63.3%)	26 (45.6%)	2.631	0.105
Respiratory symptoms	36 (73.5%)	43 (75.4%)	0.000	0.993
Gastrointestinal symptoms	27 (55.1%)	29 (50.9%)	0.057	0.811
Bleeding	18 (36.7%)	8 (14.0%)	6.159	0.013
Rash	13 (26.5%)	41 (71.9%)	19.952	<0.001
Central nervous system symptoms	18 (36.7%)	23 (40.4%)	0.033	0.856

**Table 2:** Comparison of laboratory test results between the EBV-HLH and MAS groups [n (%) or median (P25–P75)].

Factor	EBV-HLH (*n* = 49)	MAS (*n* = 57)	X^2^/Z Value	*p* Value	Adjusted *p* Value
WBC (×109/L)	2.53 (1.32–6.37)	5.30 (2.62–17.49)	−3.495	<0.001	<0.001
NEU (%)	34.20 (15.80–55.30)	68.90 (47.40–83.40)	−4.819	<0.001	<0.001
LYM (%)	56.10 (35.40–73.60)	25.40 (12.70–44.60)	4.759	<0.001	<0.001
NE (×109/L)	1.00 (0.40–1.54)	3.28 (1.27–12.29)	−5.098	<0.001	<0.001
LY (×109/L)	1.15 (0.49–3.06)	1.28 (0.86–2.08)	−0.349	0.727	0.308
HB (g/L)	98.00 (90.00–105.00)	107.00 (89.00–120.00)	−2.238	0.025	0.153
PLT (×109/L)	68.00 (45.00–138.00)	192.00 (86.00–285.00)	−4.826	<0.001	<0.001
FER (μg/L)	7795.30 (2146.20–24,833.50)	3569.70 (1726.60–8604.60)	1.885	0.059	0.024
PT (s)	14.30 (13.00–15.80)	14.00 (12.90–15.20)	1.109	0.267	0.248
APTT (s)	39.90 (35.60–44.90)	38.40 (32.10–44.60)	0.941	0.347	0.339
FIB (g/L)	1.73 (1.16–2.39)	3.65 (2.18–5.04)	−5.092	<0.001	<0.001
D-dimer (μg/L)	5100.00 (2220.00–11,750.00)	2250.00 (1280.00–9940.00)	2.424	0.015	0.009
AST (U/L)	234.90 (141.00–458.00)	104.50 (41.10–302.40)	3.238	0.001	0.002
ALT (U/L)	166.00 (66.90–255.70)	66.60 (25.20–298.00)	1.264	0.206	0.183
GGT (U/L)	140.00 (41.90–270.50)	44.90 (24.70–116.50)	2.544	0.011	0.012
LDH (U/L)	1265.50 (791.20–2431.40)	693.30 (450.40–954.90)	4.537	<0.001	<0.001
UREA (mmol/L)	3.90 (2.99–5.25)	4.34 (3.25–5.87)	−1.518	0.129	0.272
CREA (μmol/L)	31.40 (25.80–38.50)	35.60 (28.80–47.30)	−1.695	0.090	0.764
UA (μmol/L)	223.10 (188.10–290.30)	242.70 (192.00–308.50)	−1.011	0.312	0.466
TG (mmol/L)	2.46 (1.86–3.25)	1.98 (1.26–3.04)	1.838	0.066	0.117
TC (mmol/L)	3.12 (2.35–3.84)	3.91 (3.21–5.00)	−3.637	<0.001	<0.001
CRP (mg/L)	11.35 (3.23–31.48)	21.58 (4.70–75.72)	−1.876	0.061	0.047
Hemophagocytosis	36 (73.5%)	17 (29.8%)	18.369	<0.001	-

**Table 3:** Comparison of immune indicators between the EBV-HLH and MAS groups [median (P25–P75)].

Factor	EBV-HLH (*n* = 49)	MAS (*n* = 57)	X^2^/Z Value	*p* Value	Adjusted *p* Value
C3 (g/L)	1.08 (0.85–1.26)	1.14 (0.69–1.44)	−0.447	0.655	0.798
C4 (g/L)	0.40 (0.32–0.47)	0.34 (0.13–0.41)	2.977	0.003	0.002
IgA (g/L)	1.29 (0.84–1.72)	1.36 (0.96–1.95)	−0.852	0.394	0.078
IgG (g/L)	9.95 (7.26–11.91)	11.88 (8.46–17.73)	−1.835	0.067	0.459
IgM (g/L)	0.90 (0.51–1.54)	1.08 (0.81–1.45)	−1.638	0.101	0.280
CD3+%	80.98 (73.55–88.21)	72.89 (63.02–80.70)	3.292	<0.001	0.004
CD3+CD4+%	25.06 (15.08–38.50)	33.77 (24.51–41.18)	−1.850	0.064	0.039
CD3+CD8+%	41.60 (32.23–65.10)	32.08 (25.27–42.65)	2.943	0.003	<0.001
CD4+/CD8+	0.80 (0.25–1.14)	1.00 (0.66–1.51)	−2.361	0.018	0.421
CD3-CD19+%	7.90 (3.74–21.29)	15.59 (11.36–25.29)	−3.219	0.001	0.005
CD3-CD16 + CD56+%	6.00 (3.42–12.65)	7.32 (4.19–10.79)	−0.561	0.575	0.746
CD19+CD23+%	1.06 (0.40–2.50)	3.30 (1.35–6.35)	−4.176	<0.001	<0.001
CD3+ (cells/μL)	918.17 (357.40–3540.02)	918.17 (538.51–1424.00)	0.323	0.747	0.547
CD3+CD4+ (cells/μL)	367.76 (153.32–698.08)	407.19 (215.21–782.61)	−1.087	0.277	0.043
CD3+CD8+(cells/μL)	473.35 (186.93–2416.62)	420.93 (291.86–570.46)	0.944	0.345	0.781
CD3-CD19+ (cells/μL)	96.43 (31.60–216.27)	209.39 (103.81–364.52)	−3.137	0.002	<0.001
NK (cells/μL)	142.09 (25.71–255.81)	95.71 (42.05–173.64)	0.070	0.944	0.314

**Table 4:** Significant variables in univariate logistic regression.

Factor	Coefficient	Standard Error	Wald	OR	95% CI	*p* Value
Hepatosplenomegaly	−2.647	0.5	−5.29	0.071	0.027–0.189	<0.001
NEU (%)	0.042	0.009	4.505	1.043	1.024–1.062	<0.001
Rash	1.96	0.438	4.477	7.096	3.009–16.734	<0.001
LYM (%)	−0.045	0.01	−4.462	0.956	0.937–0.975	<0.001
FIB (g/L)	0.813	0.185	4.388	2.256	1.568–3.244	<0.001
Hemophagocytosis	−1.874	0.434	−4.317	0.153	0.066–0.359	<0.001
PLT (×10^9^/L)	0.01	0.002	4.017	1.01	1.005–1.015	<0.001
TC (mmol/L)	0.644	0.184	3.494	1.905	1.327–2.734	<0.001
NE (×10^9^/L)	0.303	0.09	3.365	1.354	1.135–1.615	<0.001
CD19+CD23+%	0.268	0.083	3.228	1.308	1.111–1.539	0.001
CD3+CD8+%	−0.039	0.012	−3.153	0.962	0.939–0.986	0.002
LDH (U/L)	−0.001	0	−3.139	0.999	0.998–1.000	0.002
WBC (×10^9^/L)	0.118	0.038	3.112	1.125	1.045–1.212	0.002
C4 (g/L)	−4.172	1.343	−3.106	0.015	0.001–0.215	0.002
Age	0.133	0.048	2.77	1.142	1.040–1.255	0.006
Bleeding	−1.269	0.483	−2.627	0.281	0.109–0.725	0.009
CRP (mg/L)	0.015	0.006	2.563	1.015	1.003–1.026	0.01
CD3 (g/L)	−0.039	0.016	−2.537	0.961	0.932–0.991	0.011
CD3+CD8+ (cells/μL)	0	0	−2.503	1.000	0.999–1.000	0.012
Temperature (°C)	−0.58	0.234	−2.48	0.560	0.354–0.885	0.013
GGT (U/L)	−0.003	0.001	−2.339	0.997	0.994–0.999	0.019
CD3-CD19+%	0.041	0.018	2.28	1.042	1.006–1.079	0.023
HB (g/L)	0.023	0.011	2.126	1.023	1.002–1.045	0.034
D-dimer (μg/L)	0	0	−2.119	1	1.000–1.000	0.034
CD3-CD19+ (cells/μL)	0.002	0.001	2.11	1.002	1.000–1.004	0.035
IgG (g/L)	0.069	0.034	2	1.071	1.001–1.146	0.045
FER (μg/L)	0	0	−1.964	1	1.000–1.000	0.05
CD3+ (cells/μL)	0	0	−1.962	1	1.000–1.000	0.05
Polyserositis	−0.72	0.398	−1.807	0.487	0.223–1.063	0.071
Edema	−0.653	0.413	−1.583	0.52	0.232–1.168	0.113
AST (U/L)	0	0	−1.544	1	0.999–1.000	0.123
PT (s)	−0.109	0.073	−1.497	0.897	0.778–1.034	0.134
CD3+CD4+%	0.02	0.014	1.466	1.02	0.993–1.048	0.143

**Table 5:** Multivariable logistic regression analysis of variables.

Factor	Coefficient	Standard Error	Wald	OR	95% CI	*p* Value
Rash	−2.312	0.678	11.635	0.099	0.026–0.374	0.001
FIB (g/L)	−0.812	0.301	7.260	0.444	0.246–0.802	0.007
NEU (%)	−0.037	0.014	6.903	0.963	0.937–0.991	0.009
Hemophagocytosis	1.915	0.720	7.068	6.785	1.654–27.831	0.008
Hepatosplenomegaly	1.236	0.695	3.158	3.440	0.881–13.440	0.076
Age	−0.011	0.083	0.016	0.990	0.841–1.164	0.899

**Table 6:** ROC curve analysis of core indicators in the EBV-HLH and MAS groups.

Factor	AUC	95% CI	Cut-Off	Sensitivity	Specificity	*p* Value
Combined Model	0.938	0.893–0.973	0.56	0.837	0.895	<0.001
FIB (g/L)	0.788	0.700–0.869	0.418	0.878	0.667	<0.001
Hepatosplenomegaly	0.779	0.703–0.853	0.712	0.857	0.702	<0.001
NEU (%)	0.772	0.680–0.865	0.6	0.612	0.86	<0.001
Rash	0.727	0.641–0.811	0.692	0.735	0.719	<0.001
Hemophagocytosis	0.718	0.631–0.802	0.679	0.735	0.702	<0.001

## Data Availability

The original contributions presented in this study are included in the Supplementary Materials. Further inquiries can be directed to the corresponding authors.

## Supplementary Materials

The following supporting information can be downloaded at: https://www.mdpi.com/article/10.3390/pathogens15080828/s1, File S1: the source code of the calculator (Web platforms may become invalid over time, running the risk of losing this clinical tool. To resolve this issue, we will upload the source code of the calculator together as Supplementary Materials).

## Abbreviations

EBV	Epstein–Barr virus
HLH	Hemophagocytic lymphohistiocytosis
MAS	Macrophage activation syndrome
sJIA	Systemic juvenile idiopathic arthritis
LASSO	Least absolute shrinkage and selection operator
ROC	Receiver operating characteristic
AUC	Area under the curve
DCA	Decision curve analysis
ANCOVA	Analysis of covariance
VIF	Variance inflation factor
CI	Confidence interval
OR	Odds ratio
EPV	Events per variable
WBC	White blood cell
NEU/NE	Neutrophil/Neutrophil count
LYM/LY	Lymphocyte/Lymphocyte count
HB	Hemoglobin
PLT	Platelet
FIB	Fibrinogen
FER	Ferritin
PT	Prothrombin time
APTT	Activated partial thromboplastin time
AST	Aspartate aminotransferase
ALT	Alanine aminotransferase
GGT	Gamma-glutamyl transferase
LDH	Lactate dehydrogenase
UREA	Urea
CREA	Creatinine
UA	Uric acid
TG	Triglyceride
TC	Total cholesterol
CRP	C-reactive protein
IgA/IgG/IgM	Immunoglobulin A/G/M
C3/C4	Complement 3/4
CD	Cluster of differentiation (e.g., CD3, CD4, CD8, CD19, CD23, CD56)
NK	Natural killer cells
EULAR	European League Against Rheumatism
ACR	American College of Rheumatology
PRINTO	Paediatric Rheumatology International Trials Organisation
