# Supplementary material for: Early Differential Diagnosis of Epstein–Barr Virus-Associated Hemophagocytic Lymphohistiocytosis and Macrophage Activation Syndrome in Children: A Clinical Prediction Model Based on 106 Patients
Source: Pathogens. 2026 Aug 6;15(8):828. doi: 10.3390/pathogens15080828 (PMC13516398; doi:10.3390/pathogens15080828)
Supplement: Supplementary file 1 [file pathogens-15-00828-s001.zip › pathogens-4448654-File S1.txt]

Nomogram Predictor for EBV-HLH


## EBV-HLH Diagnostic Calculator

Hepatosplenomegaly

Yes
No

Rash

Yes
No

Hemophagocytosis

Yes
No

NEU%

FIB (g/L)

Risk of EBV-HLH

-- %

\* This diagnostic calculator is based on a multivariable logistic regression model.
It is intended for clinical decision support only and should not replace professional medical diagnosis
